# Capturing CDKs in action: Live-cell biosensors pioneer the new frontiers in cell cycle research

**DOI:** 10.1247/csf.25004

**Published:** 2025-03-05

**Authors:** Sachiya Nakashima, Aika Toyama, Hironori Sugiyama, Kazuhiro Aoki, Yuhei Goto

**Affiliations:** 1 Laboratory of Cell Cycle Regulation, Graduate School of Biostudies, Kyoto University, Yoshidakonoe-cho, Sakyo-ku, Kyoto 606-8501, Japan; 2 Center for Living Systems Information Science, Graduate School of Biostudies, Kyoto University, Yoshidakonoe-cho, Sakyo-ku, Kyoto 606-8501, Japan; 3 Department of Applied Chemistry, Graduate School of Engineering, The University of Tokyo, 7-3-1, Hongo, Bunkyo-ku, Tokyo 113-8656, Japan; 4 Department of Basic Biology, School of Life Science, SOKENDAI (The Graduate University for Advanced Studies), 5-1, Higashiyama, Myodaiji-cho, Okazaki, Aichi 444-8787, Japan; 5 Division of Quantitative Biology, National Institute for Basic Biology, National Institutes of Natural Sciences, 5-1, Higashiyama, Myodaiji-cho, Okazaki, Aichi 444-8787, Japan; 6 Quantitative Biology Research Group, Exploratory Research Center on Life and Living Systems (ExCELLS), National Institutes of Natural Sciences, 5-1, Higashiyama, Myodaiji-cho, Okazaki, Aichi 444-8787, Japan

**Keywords:** CDK, FRET, cell cycle, live imaging, biosensor

## Abstract

Cyclin-dependent kinases (CDKs) orchestrate cell cycle progression through precise temporal control of substrate phosphorylation. While traditional biochemical approaches and phosphoproteomics have provided valuable insights into CDK-mediated regulation, these methods require cell population analyses and cannot capture real-time dynamics in individual cells. The recent development of fluorescent biosensors has revolutionized our ability to monitor CDK activity in living cells with unprecedented temporal and spatial resolution. Here, we comprehensively review genetically encoded fluorescent biosensors for measuring CDK activity. The two major modes of action in CDK activity biosensors—FRET-based and translocation-based biosensors—enable researchers to select appropriate tools for their specific experimental objectives. These biosensors have revealed precise spatiotemporal CDK activity dynamics across diverse model systems, including yeast, cultured mammalian cells, worms, flies, frog egg extract, fish, and mice. Such technological advances are transforming our understanding of quantitative principles underlying cell cycle control and opening new avenues for investigating cell cycle regulation in various biological contexts.

## Graphical Abstract

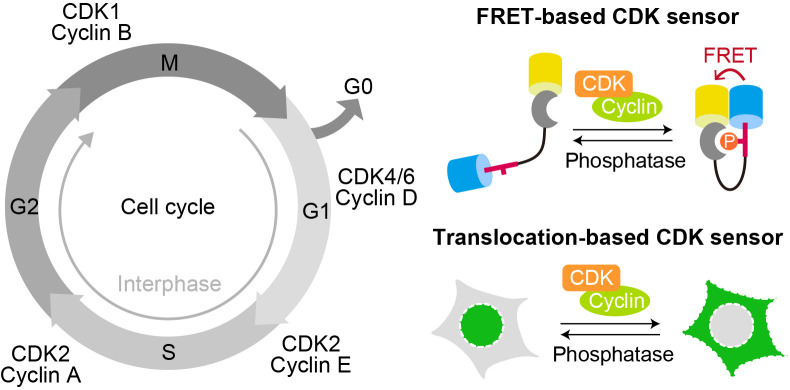

## Introduction of the Basic Principles of Cell Cycle Progression Controlled by CDK

Cell proliferation is essential for all living organisms, and the series of processes by which daughter cells arise from mother cells is called the cell cycle. The cell cycle of eukaryotes is divided into four phases; the G1, S, G2, and M phases. Cells duplicate their genome in S phase, followed by segregation of the duplicated genome, cytoplasm, and other subcellular compartments in M phase. Cell cycle progression is tightly controlled by cell cycle checkpoints, which determine the progression of cell cycle phases based on extracellular information (such as nutrient status and stress) and intracellular status (such as DNA damage) (Johnson and Walker, 1999). Since mutations in genes related to the cell cycle checkpoints could lead to congenital diseases and tumorigenesis in humans (Bury *et al.*, 2021; Malumbres and Barbacid, 2009), it is important to understand the molecular mechanisms of the cell cycle both physiologically and pathologically.

**Fig. 1:**
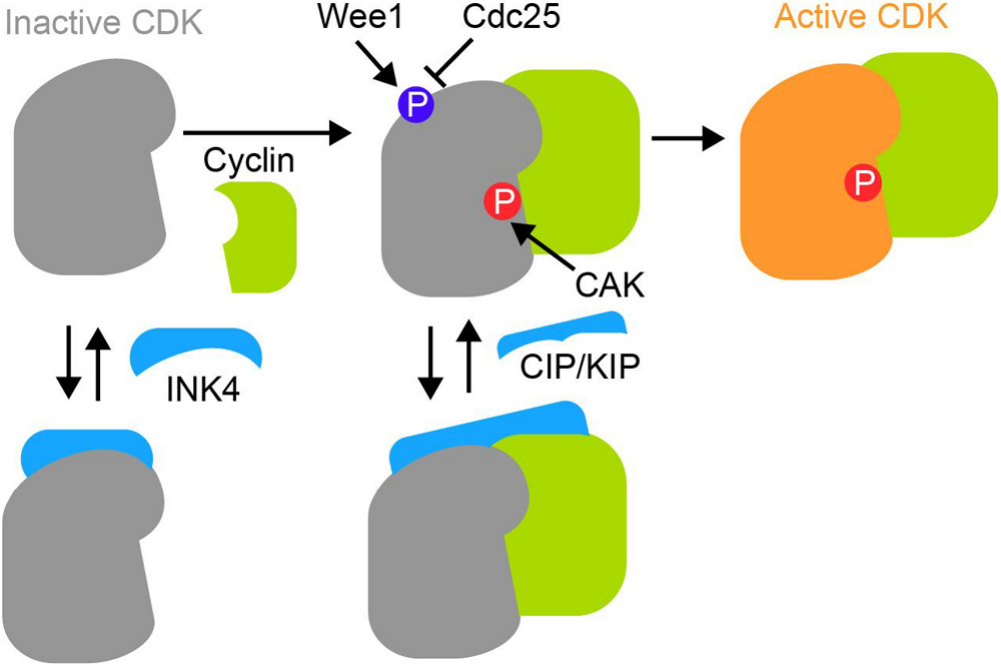
CDK activity is regulated through multiple mechanisms Cyclin-binding to CDK is a primary requirement for CDK activity. CDK activating kinase (CAK) phosphorylates the activation loop of CDK. Wee1 phosphorylates and inactivates CDK, which is counteracted by Cdc25 phosphatase. CDK inhibitors, the INK4 and CIP/KIP family proteins, directly bind CDKs and cyclins to inhibit their activity.

Cyclin-dependent kinases (CDKs) are key molecules in the progression of the eukaryotic cell cycle. Although CDKs also play pivotal roles in various cellular processes, including transcription, RNA processing, translation, development, and apoptosis, here we focus on the roles of CDKs and cyclins in the cell cycle progression. For details on the non-cell cycle functions of CDKs, please refer to other reviews (Łukasik *et al.*, 2021; Pluta *et al.*, 2023). CDKs, which are serine/threonine kinases, control various processes necessary for cell cycle progression by phosphorylating target substrates. The kinase activity of CDKs is fine-tuned by four types of regulation (Fig. 1); (1) cyclin binding, (2) phosphorylation of the CDK activation site by CDK-activating kinase (CAK), (3) phosphorylation/dephosphorylation of inhibitory phosphorylation sites of CDK, and (4) binding/dissociation by cyclin-dependent kinase inhibitors (CKIs). In the first of these regulatory processes, CDK activity is upregulated by binding to regulatory factors known as cyclins (Fig. 1). When cyclin binds to CDK, the structure of CDK changes to open the ATP binding pocket, allowing it to bind ATP. In the second process, the kinase activity of CDK is regulated by phosphorylation of CDK itself, in addition to the binding of cyclin. Specifically, CAK phosphorylates the threonine residue near the activation loop of CDK to achieve sufficient kinase activity (Fig. 1). In addition, the inhibitory phosphorylation sites, T14/Y15 of CDK1, are phosphorylated by Wee1 and Myt1 to inhibit CDK1 activity (Fig. 1). The phosphorylated T14/Y15 are dephosphorylated by the phosphatase Cdc25, leading to the activation of CDK1 and the cell cycle progression. These phosphorylation sites are conserved in CDK2, but not in CDK4/6, and at least limits CDK2 activity during replication error (Elbæk *et al.*, 2022). Finally, cyclin-dependent kinase inhibitors (CKIs) interact with monomeric CDK or cyclin-CDK complexes to suppress the kinase activity (Fig. 1). In animal cells, there are two major families of CKIs: the INK4 family and the CIP/KIP family. INK4 family proteins bind to monomeric CDKs, whereas CIP/KIP family proteins bind to cyclin-CDK complexes to inhibit CDK activity.

**Fig. 2:**
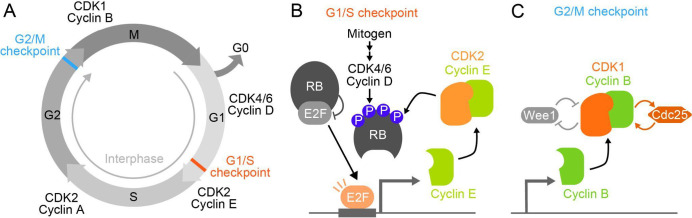
Checkpoints ensure faithful cell cycle progression (A) Cell cycle phases and CDK-cyclin complexes in mammalian cells. Different CDK-cyclin complexes execute each step of the cell cycle phases. (B) Positive feedback loop at the G1/S checkpoint of the mammalian cell cycle. Retinoblastoma protein (RB) binds to and inhibits E2F family transcription factors, and this inhibition is canceled by multiple phosphorylations on RB through the cyclin D-CDK4/6 upon the mitogen signaling. If released from phosphorylated RB, E2F activates the transcription of genes involved in cell cycle progression, including cyclin E. The cyclin E-CDK2 complex further phosphorylates RB to ensure the irreversible commitment of the cell cycle once RB phosphorylation and E2F activity rise to certain levels. (C) Double positive feedback loop at G2/M checkpoint. CDK1 activity is strictly regulated by the phosphorylation on its T14/Y15 through Wee1 kinase as well as by the accumulation of cyclin B. Wee1 is also phosphorylated and inversely inhibited by CDK1, indicating a positive feedback for CDK1. Cdc25 phosphatase counteracts Wee1 by dephosphorylating phosphorylated T14/Y15 on CDK1. It also forms a positive feedback loop between Cdc25 and CDK1. These double positive feedback loops realize the switch-like activation of CDK1 at the G2/M transition and the irreversible commitment to M phase.

The above-described regulation of CDK activity is closely linked to cell cycle checkpoints to ensure orderly cell cycle progression. In higher eukaryotes, while the expression levels of CDKs are nearly constant throughout the cell cycle, different types of cyclins are expressed in a cell cycle phase-specific manner. Thus, cell cycle phase-specific cyclin-CDK complexes phosphorylate their substrate proteins, allowing the cell cycle to progress in a timely manner (Fig. 2A). In addition, multiple positive feedbacks occur at the G1/S and G2/M transitions to achieve irreversible and discrete cell cycle progression. At the G1/S checkpoint, cyclin D accumulates in a mitogen-dependent manner and forms a complex with CDK4/6. The cyclin D-CDK4/6 complex then partially phosphorylates retinoblastoma protein RB, which inhibits E2F family transcription factors. The phosphorylation of RB releases E2Fs, promoting the gene expression of cyclin E. The accumulated cyclin E binds to CDK2, which further phosphorylates RB, forming a positive feedback loop (Bertoli *et al.*, 2013; Rubin *et al.*, 2020) (Fig. 2B). At the G2/M checkpoint, cyclin B is expressed during the G2 phase and binds to CDK1 to form a complex. In addition, CDK1 phosphorylates Wee1 and Cdc25C, which in turn activates two positive feedback mechanisms to achieve the switch-like G2/M phase progression (Deibler and Kirschner, 2010; Stewart, 2007) (Fig. 2C).

In this review, we overview methods for detecting the activity of CDKs, which play a crucial role in the cell cycle control described above, with a focus on genetically encoded biosensors for CDK activity and their applications to cell cycle studies. We also discuss the regulatory mechanisms of cell cycle checkpoints by CDKs as revealed by the genetically encoded CDK biosensors.

## Conventional Methods for Measuring CDK Activity

Traditionally, kinase activity of CDKs has been measured using biochemical techniques. Active cyclin-CDK complexes are extracted from cell lysate using either antibodies against cyclin-CDK complexes or p13^Suc1^-beads (Brizuela *et al.*, 1987; Samiei *et al.*, 1991). The kinase activity of the purified complexes is then measured by monitoring the phosphorylation of model substrates, such as human histone H1 (Martín-Castellanos *et al.*, 2000; Reynard *et al.*, 2000) or endogenous substrates (Kõivomägi *et al.*, 2021; Xiao *et al.*, 2024). This biochemical approach provides a direct measurement of the enzymatic activity of the complexes under defined experimental conditions, thereby excluding indirect effects on substrate phosphorylation. More recently, phosphoproteomic analyses have enabled the identification of comprehensive CDK substrates during the cell cycle progression (Al-Rawi *et al.*, 2023; Anders *et al.*, 2011; Chi *et al.*, 2008; Petrone *et al.*, 2016; Swaffer *et al.*, 2016; Touati *et al.*, 2018; Valverde *et al.*, 2023). Although these approaches have distinct advantages, they require cell cycle synchronization and cannot eliminate the complexity arising from cell population heterogeneity. A widely used approach for live-cell estimation of approximate CDK activity is the endogenous tagging of cyclin with fluorescent proteins such as GFP. That the resulting fluorescence can serve as a proxy for CDK activity is due to the fact that the cyclin-binding to CDK is a key rate-limiting step in CDK activation. Accordingly, cyclin accumulation roughly correlates with cell cycle progression, thereby providing an estimate of the change in CDK activity (Karuna *et al.*, 2020; Patterson *et al.*, 2019). Despite their utility for live-cell imaging, cyclin levels do not necessarily reflect actual CDK activity, which are also regulated by CDK inhibitory phosphorylation mediated by Wee1 and Cdc25, and by CDK inhibitors of the CIP and KIP families. These limitations of the conventional approaches highlight the need for direct live-cell fluorescent biosensors of CDK activity that allow visualization of temporal CDK dynamics in individual living cells. In the following sections, we summarize the currently available fluorescent biosensors for CDK activity and describe the ways they can be applied to reveal novel aspects of cell cycle regulation.

## Live-cell Imaging of CDK Activity using Genetically Encoded Fluorescent Biosensors

**Fig. 3:**
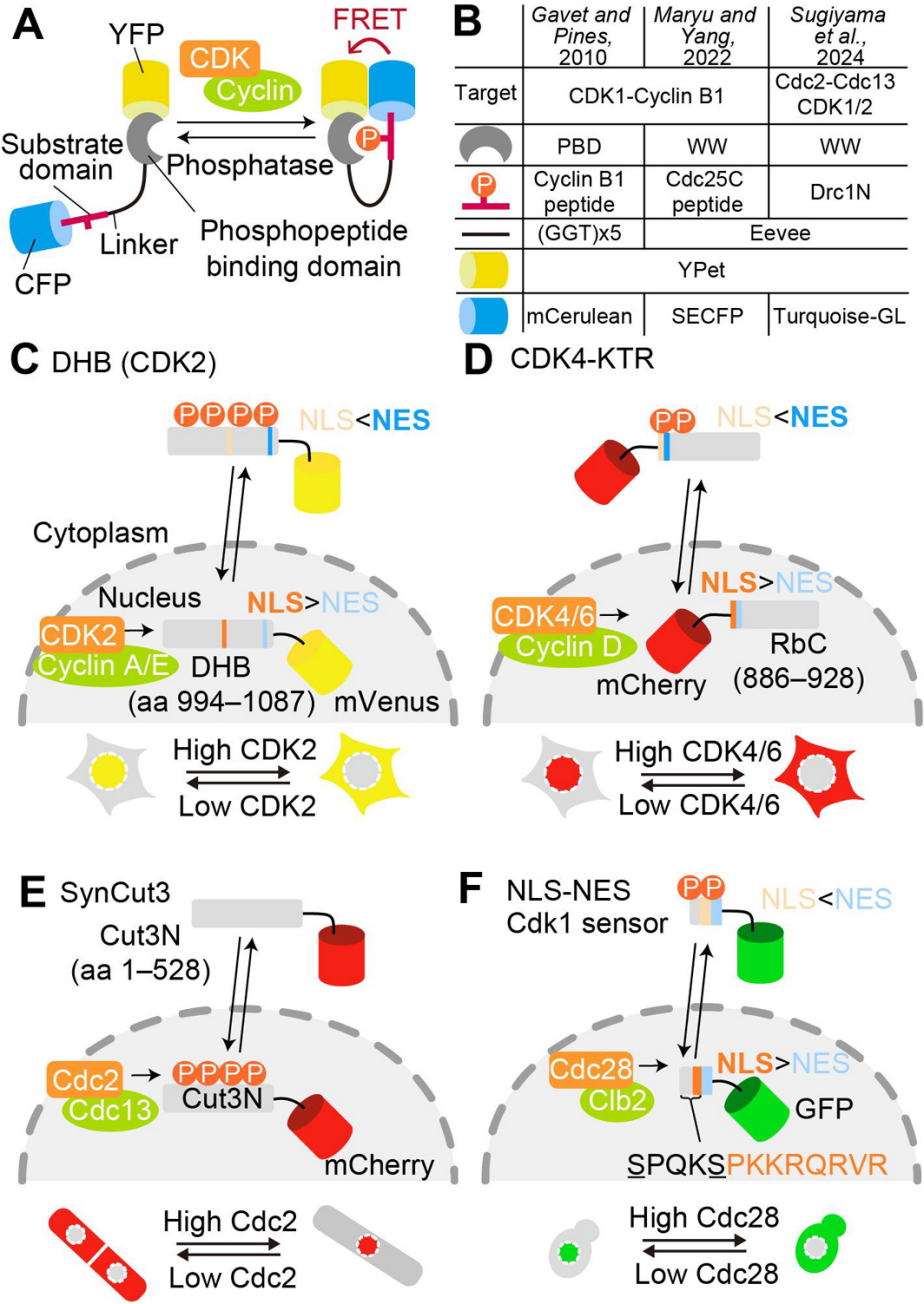
Genetically encoded fluorescent biosensors for CDK (A) Schematic illustration of the principle of the FRET-based biosensor for CDK activity. The biosensor consists of a YFP, a phosphopeptide binding domain, a linker, a substrate domain, and a CFP from the N-terminus. Once the substrate domain is phosphorylated by CDK, the phosphopeptide binding domain binds to the phosphorylated substrate domain, bringing YFP and CFP into close proximity. Dephosphorylation of a substrate domain by phosphatases alters the biosensor conformation to the open form. (B) Summary table of the previously reported FRET-based CDK biosensors. PBD: Polo-box domain from human Plk1. WW: WW domain of human PIN1. The sensor developed in Gavet and Pines, 2010 targets human CDK1 (left), the sensor developed in Maryu and Yang, 2022 targets CDK1 activity in frog eggs (middle), and the sensor developed in Sugiyama *et al.*, 2024 targets fission yeast CDK activity (right). (C) The translocation-based CDK2 biosensor for cultured mammalian cells includes a fragment of DNA helicase B (DHB), in which NLS and NES activity are regulated by CDK2-mediated phosphorylation. (D) The translocation-based CDK4/6 biosensor for cultured mammalian cells, CDK4-KTR, possesses phosphorylatable NLS and NES followed by the CDK4/6-binding domain of RB. (E) SynCut3 is based on the N-terminal fragment of condensin subunit Cut3, which is heavily phosphorylated by CDK in fission yeast cells. The phosphorylated Cut3 fragment translocates from cytoplasm to nucleus. (F) An NLS and NES module derived from budding yeast Mcm3 is used as a translocation-based Cdk1/Cdc28 biosensor.

With the advent of various fluorescent proteins and advances in fluorescence microscopy, it has become possible to detect structural changes and activity of proteins in living cells and organisms (Greenwald *et al.*, 2018). To date, kinase activity monitoring has been achieved using various kinase biosensors based on different principles (Maryu *et al.*, 2018). Here, we focus on Förster resonance energy transfer (FRET)-based biosensors and translocation-based biosensors for measuring CDK activity. Förster resonance energy transfer (FRET)-based biosensor is a promising strategy to visualize phosphorylation events through the change in fluorescence intensity from donor and acceptor fluorescent proteins (Goto *et al.*, 2021). Most of the reported FRET biosensors for kinases are classified as intramolecular FRET biosensors, which contain donor and acceptor fluorescent proteins such as CFP and YFP, respectively (Maryu *et al.*, 2018). Further, these fluorescent proteins sandwich a phosphopeptide-binding domain, linker, and substrate domain (Fig. 3A). These FRET biosensors are designed to be phosphorylated by target kinases and dephosphorylated by phosphatases, thereby monitoring the balance between target kinases and phosphatases.

Gavet and Pines developed the first FRET biosensor for CDK activity (Gavet and Pines, 2010); their biosensor targeted human CDK1 and was later improved (Belal *et al.*, 2014). The Polo-Box Domain from human Plk1 (a.a. 373–592) and a peptide from cyclin B1 (a.a. 117–132) containing an autophosphorylated S126 residue serve as the phosphopeptide-binding domain and substrate domain, respectively (Fig. 3B). Upon phosphorylation, the substrate peptide is intramolecularly bound to the phosphopeptide-binding domain, bringing YPet and mCerulean into close proximity and increasing FRET efficiency. This biosensor has successfully enabled the visualization of a steep increase in CDK1 activity at the onset of M phase and its subsequent drop at the end of M phase in cultured mammalian cells (Gavet and Pines, 2010). Due to the conserved substrate specificity of CDK1 between species, this biosensor has been utilized in *Drosophila* embryos as well as in mammalian cultured cells (see below).

Recently, the demand for more sensitive CDK activity biosensors has led to the development of new intramolecular FRET biosensors targeting *Xenopus* egg extracts and fission yeast (Fig. 3B). These FRET biosensors rely on the optimal backbone for intramolecular FRET biosensors, namely the Eevee backbone (Komatsu *et al.*, 2011). The Eevee backbone includes a long flexible linker called the Eevee linker, thereby reducing the effect of the relative angle between donor and acceptor fluorophores and rendering biosensors distance-dependent. Further, dimerization-prone fluorescent protein pairs such as YPet and SECFP enhance the gain of FRET increase in the Eevee backbone-based FRET biosensors. By using the Eevee backbone, Maryu and Yang developed a CDK1 activity FRET biosensor derived from EKAREV (Komatsu *et al.*, 2011), a FRET biosensor for ERK MAP kinase activity (Maryu and Yang, 2022). The substrate domain of the original EKAREV contains a peptide derived from Cdc25C, which is phosphorylated by both CDK1 and ERK, and the ERK binding sequence FQFP in order to render it more sensitive to ERK than CDK1 (Gonzalez *et al.*, 1991; Harvey *et al.*, 2008). To obtain a FRET biosensor for CDK1 activity, the authors removed the ERK binding sequence FQFP and succeeded in visualizing the dynamics of CDK1 activity in *Xenopus* egg extract.

Our group recently developed an intramolecular FRET biosensor for the fission yeast *Schizosaccharomyces pombe* (Fig. 3B) (Sugiyama *et al.*, 2024). We also employed the Eevee backbone to design a FRET biosensor for CDK1 activity (Komatsu *et al.*, 2011). In addition, we used a nuclear localization sequence (NLS) at the C-terminus of the biosensor, because CDK is predominantly localized in the nucleus throughout the cell cycle in fission yeast (Decottignies *et al.*, 2001). After extensive screening for an optimal substrate domain from among candidate CDK substrates identified in previous phosphoproteomic studies (Swaffer *et al.*, 2016), we finally found that the N-terminal domain of Drc1 (35–190 a.a., Drc1N) serves as a good substrate for CDK1, and we designated the resulting biosensor Eevee-spCDK. Drc1 is a DNA replication checkpoint protein that plays a crucial role in the stress response during S phase and is phosphorylated by CDK during normal cell cycle progression (Fukuura *et al.*, 2011; Noguchi *et al.*, 2002). Drc1N contains ten possible phosphorylation sites by CDK1, and therefore Eevee-spCDK is sensitive enough to detect CDK1 activity in S, G2, and M phases. Indeed, Eevee-spCDK showed an approximately 40% increase in FRET efficiency in a cyclin concentration-dependent manner. Because human CDK1 functionally compliments fission yeast CDK1 homolog Cdc2 (Lee and Nurse, 1987), Eevee-spCDK allows monitoring CDK1/2 activity in mammalian cultured cells, suggesting the potential applications of Eevee-spCDK across different organisms. One advantage of FRET-based CDK biosensors is their ability to detect subcellular CDK activity such as the CDK activity of fission yeast nuclei with NLS (Sugiyama *et al.*, 2024). The analysis of localized CDK activity in the centrosome, kinetochores, and cytoplasm represents a promising direction for future research. However, these biosensors have certain limitations; they require specialized FRET-imaging equipment, and occupy a wider wavelength range because of the two fluorescent proteins, limiting multiplexed imaging.

Translocation-based biosensor is an alternative tool for visualizing CDK activity in living cells. The first translocation-based CDK biosensor was developed by the Meyer Lab for monitoring CDK2 activity in cultured mammalian cells; it consists of the C-terminal region of human DNA helicase B (DHB) fused to a fluorescent protein (Spencer *et al.*, 2013) (Fig. 3C). This DHB fragment contains four CDK phosphorylation sites flanked by a NLS, with a nuclear export signal (NES) at the C-terminal end. CDK2 phosphorylates these sites and alleviates the NLS activity, resulting in translocation of the CDK2 biosensor from the nucleus to the cytoplasm (Fig. 3C). These phosphorylations are primarily mediated through CDK2 in complexes with cyclin E and cyclin A (Spencer *et al.*, 2013), and to lesser extent through cyclin E1- and cyclin A2-CDK1 complexes (Schwarz *et al.*, 2018). Additional details about this biosensor are available (Martinez and Matus, 2022).

After that, nuclear-cytoplasmic shuttling has proven particularly effective for visualizing kinase activity, as demonstrated by the kinase translocation reporters (KTR) (Regot *et al.*, 2014). In general, KTRs include kinase docking domain, bipartite NLS, NES, and fluorescent protein, and phosphorylation within the NLS and NES by its intended kinase changes the balance between NLS and NES. The CDK4/6 activity biosensor for cultured mammalian cells has been developed by taking advantage of KTR technology (Regot *et al.*, 2014; Yang *et al.*, 2020) (Fig. 3D). This reporter contains a short C-terminal fragment of retinoblastoma (RB) protein (a.a. 886–928) as a cyclin D-CDK4/6 docking domain, which is required for binding and phosphorylation of RB by CDK4/6, but not by CDK2 (Topacio *et al.*, 2019; Wallace and Ball, 2004). The mode of action of this biosensor is similar to that of the DHB-based CDK2 biosensor, and therefore, by using different fluorescent proteins, multiplexed imaging of CDK2 and CDK4/6 activity can be achieved at the single cell level (Yang *et al.*, 2020).

To monitor fission yeast CDK activity, the Nurse group has developed SynCut3, a synthetic design of the fission yeast Cut3-based CDK biosensor (Patterson *et al.*, 2021). The fission yeast Cut3, a homolog of SMC4 (an SMC subunit of condensin), translocates from the cytoplasm to the nucleus upon CDK-dependent phosphorylation of a single site in its N-terminus (Sutani *et al.*, 1999). SynCut3 contains the first 528 amino acids of Cut3, a region sufficient for translocation at mitosis (Fig. 3E). Due to its sensitivity as a substrate, SynCut3 predominantly detects CDK activity during M phase (Swaffer *et al.*, 2016). Note that the translocation pattern of SynCut3 is opposite to that of other translocation-based CDK biosensors; SynCut3 accumulates at the nucleus upon phosphorylation. For budding yeast *Saccharomyces cerevisiae*, a translocation-based CDK1/Cdc28 biosensor has been developed based on phospho-regulated NLS fused to an NES (Örd *et al.*, 2019). The regulatory domain of this biosensor is derived from CDK phosphorylation sites in Mcm3, a subunit of the Mcm2-7 complex (Liku *et al.*, 2005). Similar to mammalian CDK2 and CDK4/6 biosensors, the budding yeast CDK1 biosensor translocates from the nucleus to the cytoplasm upon CDK-mediated phosphorylation (Fig. 3F).

Translocation-based biosensors can be readily implemented with conventional microscope equipment in comparison to FRET-based biosensors. As demonstrated by Yang *et al.*, 2020, multiplexed imaging using translocation-based biosensors, such as simultaneous imaging of CDK2 and CDK4/6 activity in a living cell, is feasible because it requires only a single fluorophore. However, it is inherent to the design of translocation-based biosensors that they are incapable of visualizing the subcellular localization of CDK activity, e.g., CDK activity at the plasma membrane or at the spine of neuronal dendrites. Additionally, it is impossible to monitor CDK activity during M phase in mammalian cells because of the nuclear envelope breakdown. In the following sections, we summarize the current applications of these biosensors in different organisms including yeast, cultured cells, and animals.

## Live-cell Imaging of CDK Activity Provides Direct Evidence of a Threshold at G2/M Transition In Fission Yeast

**Fig. 4:**
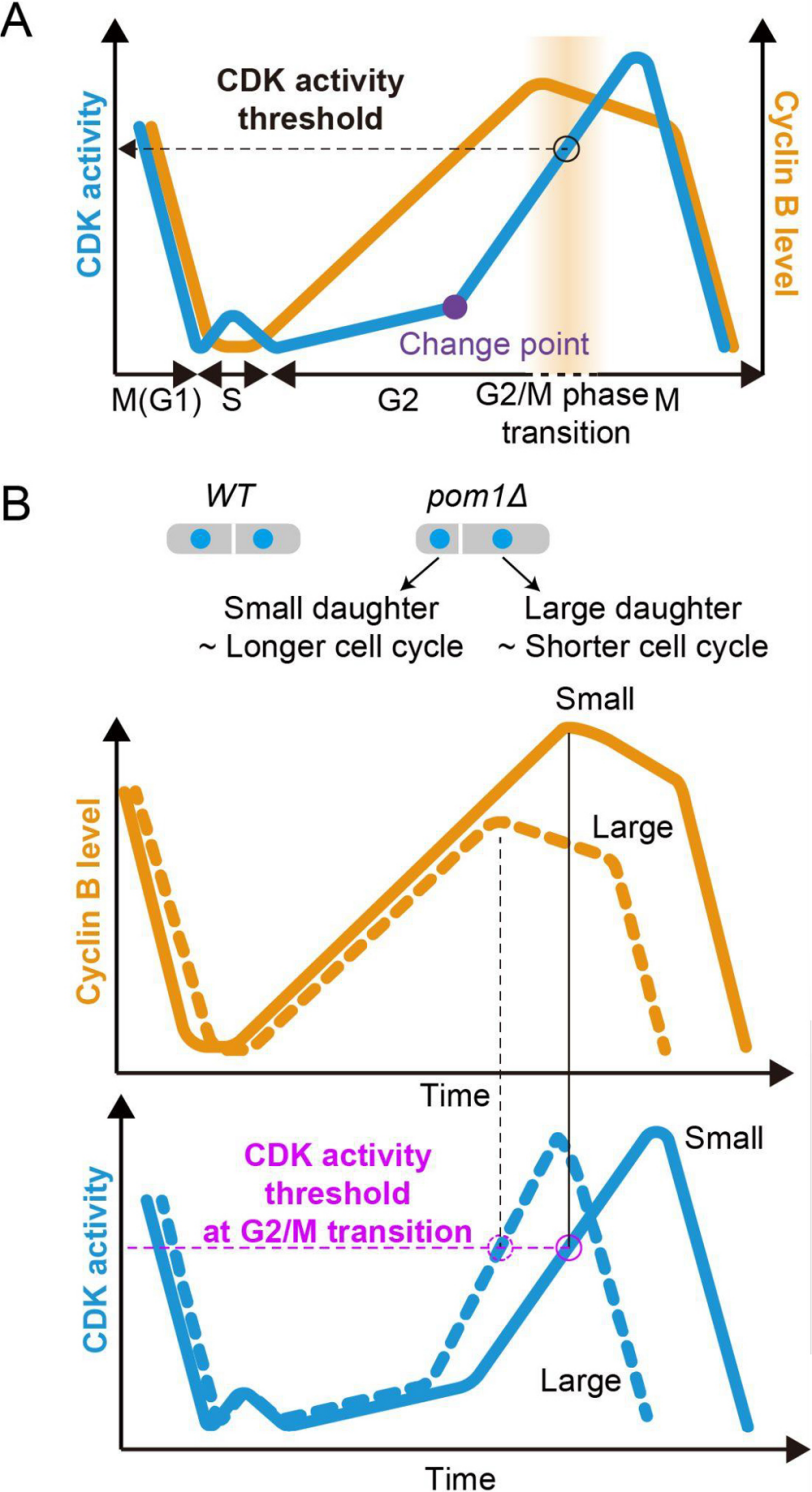
CDK dynamics in fission yeast and robust CDK activity threshold at G2/M transition (A) CDK activity exhibits distinctive characteristics: a transient peak at S phase, change point at late G2, robust threshold at G2/M, and rapid decrease at the end of M phase. (B) Different sizes of daughter cells can be obtained in the *pom1Δ* strain. Difference in the birth cell size results in the difference in cell cycle duration due to the homeostasis of cell size. Therefore, small daughters tend to spend longer time for division, resulting in higher accumulation of cyclin at division. Despite the different levels of cyclin accumulation between small and large daughter cells, CDK activity levels at G2/M transition become uniform. This robust threshold of CDK activity might be achieved by the integration of several regulatory mechanisms including Wee1-Cdc25.

The fission and budding yeasts have been extensively used to elucidate the core principles of cell cycle mechanisms. These model organisms offer many advantages for studying the cell cycle due to their genetic simplicity, particularly their limited number of genes encoding CDKs and cyclins. In the fission yeast, only a single cell cycle CDK, *cdc2*, is present (Nurse and Bissett, 1981; Nurse *et al.*, 1976). Furthermore, among the six cyclin-encoding genes, only *cdc13* is indispensable for cell growth (Booher and Beach, 1988; Booher *et al.*, 1989; Coudreuse and Nurse, 2010; Martin-Castellanos *et al.*, 1996; Moreno *et al.*, 1989), indicating that the single cyclin-CDK complex suffices to control ordered cell cycle events. This minimal cell cycle regulatory network implies the need for a “quantitative model” of cell cycle regulation, suggesting that cell cycle progression is determined by the timing of CDK activity at certain thresholds (Basu *et al.*, 2022a; Coudreuse and Nurse, 2010). Recent time-resolved phosphoproteomic analysis has suggested the existence of different phosphorylation thresholds for distinct substrates (Swaffer *et al.*, 2016), providing a mechanistic basis for the ability of a single cyclin-CDK complex to coordinate all cell cycle events across distinct cell cycle stages. This quantitative model presumes that CDK activity progressively increases during cell cycle progression, sequentially passing through certain thresholds to trigger corresponding events in the next cell cycle through substrate-specific phosphorylation. In addition, mathematical models of the fission yeast cell cycle have predicted the existence of a critical threshold at which CDK activity bursts through a double-positive feedback-loop involving Wee1 and Cdc25 (Novak *et al.*, 1998; Novak and Tyson, 1997), but direct observation of CDK activity dynamics has remained elusive. Recently, our group developed a FRET biosensor for CDK activity, designated as Eevee-spCDK (Sugiyama *et al.*, 2024), which enables real-time monitoring of CDK activity in individual fission yeast cells. By using Eevee-spCDK in fission yeast, several characteristic features of CDK activity dynamics were revealed, including a transient peak at S phase, a distinct change point in late G2 phase, a robust threshold at G2/M transition, and a rapid decrease at the end of M phase (Fig. 4A). The knockout of the non-essential cyclins *cig1*, *cig2*, and *puc1* abolished the transient CDK activity peak in S phase, which is consistent with time-resolved phosphoproteomic analysis (Swaffer *et al.*, 2016). This suggests that the qualitatively distinct cyclin-CDK complexes play specific roles to exert an effective and faithful S phase progression. The change point, which delineates the biphasic increase in CDK activity at the late G2 phase, is another key characteristic feature of the CDK dynamics revealed by this biosensor. The two positive feedback-loops mediated by Wee1 and Cdc25 have been proposed to provide this biphasic pattern of CDK activity, generating bistability in the cell cycle system (Gérard *et al.*, 2015; Novak *et al.*, 1998; Sveiczer *et al.*, 2000). Although the prevailing quantitative model postulates the existence of CDK activity thresholds at cell cycle transition, direct measurement of such thresholds has remained elusive. The fission yeast cells lacking *pom1*, in which asymmetric cell division takes place and produces daughter cells with different cell sizes, exhibit uncoupling between cyclin levels and CDK activity, while the cells demonstrate a robust threshold of CDK activity at the G2/M phase transition independent of initial cell size and cell cycle duration (Fig. 4B). In consideration of all the above, Eevee-spCDK has provided both the direct visualization of predicted characteristics of CDK activity dynamics and definitive evidence supporting the significance of CDK activity threshold at G2/M transition.

An alternative CDK activity biosensor for fission yeast, SynCut3, which relies on the phosphorylation-dependent protein translocation, has been developed and utilized in multiple studies (Patterson *et al.*, 2021). Based on the characteristics of phosphorylation of Cut3 by CDK (Sutani *et al.*, 1999; Swaffer *et al.*, 2016), SynCut3 predominantly detects CDK activity during M phase. Because of its ease of use, high signal-to-noise ratio, and phase-specificity, SynCut3 serves as an effective M phase marker, enabling comparison of detailed CDK activity patterns in M phase, and multiplexed imaging with cyclin-CDK complex levels (Basier and Nurse, 2023; Basu *et al.*, 2022a, 2022b; Murciano-Julià *et al.*, 2025; Novák and Tyson, 2024; Patterson *et al.*, 2021). In addition, the combination of two translocation-based biosensors with different sensitivity has unveiled an activation-lag of CDK between the cytoplasm and nucleus (Kapadia and Nurse, 2024).

## Rewiring the Classical View of Cell Cycle Commitment in Mammalian Cells

**Fig. 5:**
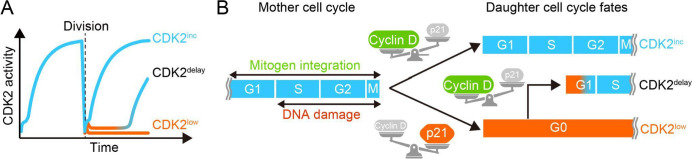
The competing integration of mitogen signaling and DNA damage response in the mother cell determines subsequent CDK2 activity patterns and cell fates in daughter cells (A) Following mother cell mitosis, CDK2 activity bifurcates, either showing rapid increase (CDK2^inc^) or remaining at low levels (CDK2^low^). A subset of CDK2^low^ cells subsequently recover CDK2 activity (CDK2^delay^). (B) The decision between cell cycle commitment or exit in daughter cells is primarily determined by the balance between integrated mitogen signaling and DNA damage response in the mother cell. Mitogen signaling initiates cyclin D synthesis, while DNA damage activates the p53 pathway, leading to p21 synthesis. The cyclin D-CDK4/6 complex promotes cell cycle commitment, while p21 inhibits CDK activity, resulting in cell cycle exit.

A series of groundbreaking papers, starting with Spencer *et al.*, 2013, have revolutionized our understanding of the restriction point. The restriction point was classically believed to exist in mid-G1 phase, where cells decide whether to proliferate or stay quiescent according to mitogen signaling (Pardee, 1974). A DHB-based CDK2 biosensor expressed in non-transformed human mammary epithelial cell line MCF-10A exhibits a bifurcation very early in the G1 phase of daughter cells, which corresponds to their subsequent cell fate (Fig. 5A) (Spencer *et al.*, 2013). Importantly, this bifurcation of CDK activity is already dictated at G2 phase of mother cells by the integration of mitogen signaling (Min *et al.*, 2020; Yang *et al.*, 2017) and DNA damage from an endogenous replication stress (Arora *et al.*, 2017; Daigh *et al.*, 2018). At the molecular level, these extrinsic and intrinsic signals are converted into the balance between cyclin D and p21, a CIP/KIP family CDK inhibitor activated through the p53 pathway (Fig. 5B). When cyclin D is more abundant than p21, CDK4/6 activity increases followed by the initiation of a cyclin E-CDK2 positive feedback loop (Chung *et al.*, 2019; Gookin *et al.*, 2017; Kim *et al.*, 2022; Moser *et al.*, 2018; Overton *et al.*, 2014; Spencer *et al.*, 2013; Yang *et al.*, 2020). These important discoveries have taken full advantage of live-cell visualization of CDK activity using fluorescent biosensors.

## CDK Activity Measurements in Metazoa Unveils the Key Roles of CDK in Development

### CDK governs cell fate decisions in *C. elegans*

In *C. elegans*, the DHB-based CDK biosensors have been successfully implemented and have revealed new aspects of developmental cell cycle regulation. Similar to observations in mammalian cultured cells, CDK activity levels shortly after mitosis can predict whether cells will continue cycling or enter quiescence in *C. elegans* development (Adikes *et al.*, 2020). The integration of the DHB-based CDK biosensor with other fluorescent reporters has further enhanced our understanding of cell cycle regulation. Specifically, combining CDK biosensors with PCNA markers has enabled precise determination of S-phase entry (van Rijnberk *et al.*, 2017), revealing cell cycle phase-specific cell exclusion (Dwivedi *et al.*, 2021). Furthermore, the CDK biosensor has uncovered unexpected plasticity in previously characterized invariant cell lineages, particularly in the context of temperature-dependent variation in vulval development (Adikes *et al.*, 2020). *C. elegans* possesses a highly definitive cell-lineage, particularly in vulva development, which is spatiotemporally regulated (Sternberg and Horvitz, 1986). In this process, the D cell typically halts its cell cycle and undergoes differentiation. However, under high temperature conditions, the cell fate of the D cell during vulva development becomes stochastically altered, leading to continued cell cycle progression. Cells that continue cycling exhibit progressively increasing CDK activity after birth, which is potentially regulated by fluctuations in the activity of the CDK inhibitor, CKI-1. This finding implies the presence of cryptic plasticity in supposedly invariant cell-lineages in response to environmental cues. Additionally, the application of the CDK biosensor has challenged the traditional view regarding the requirement of cell cycle exit for terminal differentiation. The anchor cell (AC) invasion in *C. elegans* serves as an excellent *in vivo* model for studying the regulation of cell invasion during development. The AC normally halts its cell cycle before initiating invasion, a process predominantly triggered by a decrease in CDK activity through cell cycle regulators (Martinez *et al.*, 2024). However, when cell cycle regulators are perturbed in the AC, cells can maintain their differentiated functions while continuing to proliferate with progressively increasing CDK activity (Martinez *et al.*, 2024). This observation challenges the traditional dichotomy between proliferation and differentiation. Specifically, it demonstrates that cells can maintain specialized functions and differentiated states while actively cycling, contrary to the conventional view that terminal differentiation requires permanent cell cycle exit. This finding suggests a more nuanced relationship between cell cycle activity and differentiation than previously appreciated.

### Propagation of CDK1 activation waves drives the rapid and synchronous cell division in fertilized eggs of *Drosophila* and *Xenopus laevis*

During organismal development, rapid cell proliferation, differentiation, tissue growth, and morphogenesis occur, and precise regulation of the cell cycle is crucial for these events. *Drosophila*, *Xenopus laevis*, zebrafish, and mice are major model organisms in developmental biology, and several studies have investigated the relationship between developmental events and CDK activity in these models. During the early stages of egg cleavage in some organisms such as *Drosophila* and *Xenopus laevis*, cell divisions often occur rapidly and synchronously, utilizing cell cycle regulatory factors stored in the egg cytoplasm, independently of zygotic genome activation. However, the size of the eggs substantially exceeds that of somatic cells, with *Drosophila* embryos measuring about 0.5 mm (Foe and Alberts, 1983) and *Xenopus* embryos measuring about 1.2 mm (Dumont, 1972). Simple diffusion of cell cycle regulatory factors cannot explain the propagation of such spatially synchronized mitosis. Chemical waves, which can propagate faster and farther than simple diffusion waves, are proposed to drive mitotic waves in the cytoplasm of such large eggs (Novak and Tyson, 1993). There exist two types of chemical waves: trigger waves and phase waves. Trigger waves propagate through a combined effect of diffusion and local reactions, while phase waves require neither spatial synchronization nor diffusion. Observations in *Xenopus* egg extracts have demonstrated that trigger waves drive mitotic waves (Chang and Ferrell, 2013). Researchers have employed biosensors to investigate the mechanisms of mitotic synchronization and fast propagation of mitotic waves through direct visualization of CDK1 activity.

**Fig. 6:**
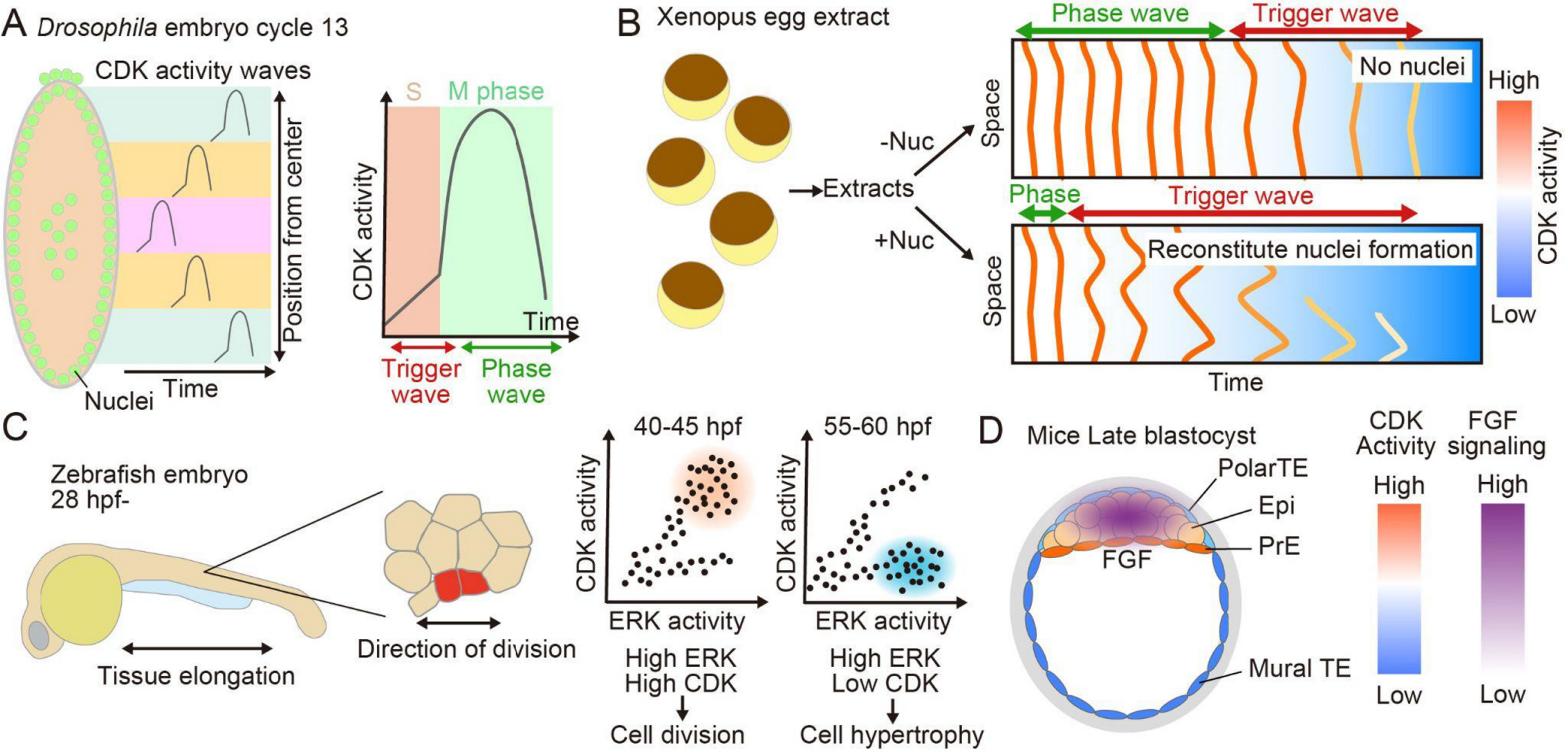
CDK activity during development in metazoa (A) Spatiotemporal waves of CDK1 activity in *Drosophila* embryos. During cycle 13, CDK activity exhibits biphasic waves, comprising trigger waves during S phase and phase waves during M phase. Chk1 activity regulates the propagation speed of CDK activity waves. (B) CDK waves in *Xenopus* egg extracts. The reconstitution of nuclear formation by the addition of sperm DNA accelerates the transition of CDK activity waves from phase waves to trigger waves. (C) Cell division-dependent periderm elongation in zebrafish embryos starting at 24 hpf. During anterior–posterior (A–P) axis elongation of the tailbud periderm, cell divisions occur along the A–P axis. A proportional relationship exists between the frequency of mitosis and tissue elongation. Elongation speed decreases over time, potentially regulated by alterations in CDK responsiveness to the EGF-ERK pathway. (D) Lineage-specific CDK activity levels in late blastocyst mouse embryos. While the epiblast (Epi) and primitive endoderm (PrE) exhibit high CDK activity, the trophectoderm (TE)—especially the mural TE—exhibits low activity. An FGF signaling gradient may underlie these differential CDK activity levels.

Implementation of a FRET biosensor for CDK1 activity (Gavet and Pines, 2010) revealed that biphasic waves of CDK1 activity propagate along the anterior–posterior axis of *Drosophila* embryos during S phase and M phase, presumably driving mitotic waves (Fig. 6A) (Deneke *et al.*, 2016). Although it was initially assumed that trigger waves alone controlled the mitotic waves, visualization of CDK activity revealed that both trigger and phase waves contribute to mitotic waves (Deneke *et al.*, 2016). As the cell cycle duration elongates approaching maternal–zygotic transition, mitotic waves slow down in a Chk1-dependent manner, which regulates the DNA replication checkpoint (Deneke *et al.*, 2016; Vergassola *et al.*, 2018). While positive feedback mediated by Wee1 and Cdc25 is necessary for rapid activation of CDK1, it does not control the propagation speed of CDK1 activity (Deneke *et al.*, 2016). Nuclear positioning within the embryo is crucial for the synchronization of mitosis before maternal-zygotic transition, whereby trigger waves emerge instead of phase waves when nuclear density is altered (Deneke *et al.*, 2019; Hayden *et al.*, 2022).

The development of a FRET biosensor for CDK1 activity has enabled the observation of biphasic CDK1 activation in *Xenopus* egg extracts (Maryu and Yang, 2022). Nuclear compartmentalization of cell cycle regulators ensures robust mitotic timing. Furthermore, spatial heterogeneity within the cell, including nuclear compartmentalization, plays a critical role in facilitating the rapid propagation of mitotic waves by accelerating the transition from faster phase waves to trigger waves that can propagate over extended distances (Fig. 6B) (Puls *et al.*, 2024). Consequently, visualization of CDK1 activity has significantly advanced our understanding of the mechanisms underlying fast-synchronized cell division-propagation in early development.

### CDK activity involvement in cell fate decision and tissue growth in vertebrate development

The extracellular signal-regulated kinase (ERK) pathway governs cell proliferation and differentiation in vertebrate developmental processes (Nakamura *et al.*, 2021) through its interaction with CDK activity. As a model system of vertebrate development, the DHB-based CDK2 biosensor (Spencer *et al.*, 2013) has been introduced into zebrafish embryos (Adikes *et al.*, 2020). In the tailbud of zebrafish embryos, CDK activity rapidly decreases just before anaphase, and post-anaphase CDK activity profiles are segregated into two populations—those that increase in activity and those that maintain a low level of activity. These CDK activity patterns differ according to cell fate; for example, notochord progenitor cells and primitive red blood cells in the intermediate cell mass display high CDK activity, while adaxial cells exhibit low CDK activity at 24 hours post-fertilization (hpf). Recent studies have also explored the relationship between the anterior-posterior (A–P) axis elongation of zebrafish embryonic periderm and CDK activity (Fig. 6C) (Ramkumar *et al.*, 2024). This elongation initiates post-gastrulation, peaks at 24–48 hpf, and continues at a slower rate throughout embryonic development. While the whole embryo undergoes greater increases in length than width, the periderm exhibits similar elongation between its length and width. A proportional relationship exists between the periderm elongation rate and the frequency of cell divisions, with a bias in cell division orientation towards the A–P axis. Simultaneous monitoring of CDK and ERK activity using the DHB-based CDK2 biosensor (Spencer *et al.*, 2013) and ERK-KTR (Regot *et al.*, 2014) demonstrated that the population of cells exhibiting high CDK and high ERK activity decreased from 40–45 hpf to 55–60 hpf, while cells with high ERK activity and low CDK activity increased. ERK signaling also regulates cell hypertrophy, which occurs more frequently after 52 hpf than before. This suggests that elongation speed is regulated by changes in proliferative and hypertrophic responsiveness to ERK signaling during later developmental stages.

The differentiation of inner cell mass (ICM) and trophectoderm (TE) represents the first cell fate decision in mouse embryonic development. Analysis using the DHB-based CDK2 biosensor (Spencer *et al.*, 2013) has revealed that CDK activity remains high in all cells until the mid-blastocyst stage, and subsequently declines in TE cells, especially in mural TE cells (Fig. 6D). This suppression of CDK activity in the TE results from the establishment of an FGF4-ERK signaling gradient along the embryonic-abembryonic axis, with high levels maintained on the ICM side and low levels present on the TE side (Azami *et al.*, 2019; Christodoulou *et al.*, 2019; Guo *et al.*, 2010; Nowotschin *et al.*, 2019; Simon *et al.*, 2020). However, CDK activity remains comparable between GATA-positive PrE and NANOG-positive Epi cells in the late blastocyst stage (Saykali *et al.*, 2024), indicating that cell fate is not solely determined by changes in CDK activity but rather governed by temporal dynamics of ERK signaling in ICM differentiation (Pokrass *et al.*, 2020). Together, these findings indicate that spatiotemporal regulation of CDK activity by ERK signaling is a key mechanism in multiple developmental events, although ERK signaling does not simply activate CDK but rather properly separates proliferation and differentiation by unknown mechanisms.

## Limitations and Future Perspectives of CDK Activity Biosensors

As summarized in this review, the development of fluorescent CDK activity biosensors has revealed new underlying regulatory mechanisms of cell cycle control systems through spatiotemporal regulation of CDK activity. Current research using these biosensors faces several limitations and future challenges that need to be addressed. The most significant limitation lies in biosensor specificity. While specificity against non-CDK kinases has been established, different cyclin-CDK complexes can share substrates due to their relatively flexible phosphorylation specificity, making it difficult to precisely determine which cyclin-CDK complex is responsible for the CDK activity being observed. Furthermore, we must consider that each biosensor is designed based on a single model substrate, and its phosphorylation dynamics may not necessarily correspond to the phosphorylation dynamics of all substrates within the cell. While this limitation is inherent to all reporter systems, CDK-based systems may be particularly susceptible to discrepancies between model substrates and the phenomena of interest because some substrates have different sensitivities to CDK activity. To investigate the detailed molecular mechanisms of cell cycle regulation by CDK, live cell imaging of CDK activity requires a complementary approach such as phosphoproteomics.

One promising future research direction is the observation of CDK activity in non-canonical cell cycles. Meiosis represents a prime example, where two consecutive cell divisions occur without an intervening DNA replication phase, requiring incomplete reduction of CDK activity at the end of M phase (Izawa *et al.*, 2005). How CDK is regulated in this cell cycle, which differs entirely from mitosis, is not yet fully understood. Additionally, recent tracking of cell divisions in developing organisms has revealed various non-canonical division modes, such as DNA replication-independent cell division in developing zebrafish epidermis (Chan *et al.*, 2022) and nuclear division-independent cytokinesis in developing flies (Bakshi *et al.*, 2023). Live imaging of CDK activity in organisms is expected to facilitate the discovery and molecular mechanism elucidation of these diverse cell division modes. The future development of more sophisticated biosensors—including those with enhanced sensitivity, improved spatial resolution, expanded dynamic range, and capabilities for simultaneous monitoring of multiple parameters—, combined with emerging imaging technologies and analytical methods, promises to further illuminate the complex spatiotemporal dynamics of cell cycle regulation in living systems.

## Author Contributions

Conceptualization, Writing – Original Draft: S.N., A.T., K.A., Y.G.; Writing – Review & Editing: S.N., A.T., H.S., K.A., Y.G.; Funding Acquisition: K.A., Y.G.

## Fundings

K.A. was supported by JSPS KAKENHI grants (nos. JP22H02625, JP24H01416, and JP24K21981), NAGASE Science Technology Foundation, Takeda Science Foundation, the grant of Joint Research by the National Institutes of Natural Sciences (NINS) (NINS program No. OML012404), Joint Research of the Exploratory Research Center on Life and Living Systems (ExCELLS) (ExCELLS program No. 23EXC601), and the Cooperative Research Program (Joint Usage/Research Center program) of Institute for Life and Medical Sciences, Kyoto University. Y.G. was supported by a JST, ACT-X grant (no. JPMJAX22B8), and JSPS KAKENHI grants (nos.19K16050 and 22K15110). H.S. was supported by JSPS KAKENHI grants (nos. 21J01354 and 22K15115) and the Dr. Yoshifumi Jigami Memorial Fund, The Society of Yeast Scientists.

## Conflict of Interest

The authors declare no competing financial interests.
